# 1489. CD4+ T Cell Count and Associated Mortality in PLWHIV and Cancer at an Oncologic Center in Mexico over a 10-year Period.

**DOI:** 10.1093/ofid/ofad500.1324

**Published:** 2023-11-27

**Authors:** Alejandro Olmedo-Reneaum, P A T R I C I A VOLKOW-FERNÁNDEZ, N A N C Y MARTÍNEZ-RIVERA, M A R ÍA J O S É MENDOZA-PALACIOS, B E D A ISLAS-MUÑOZ

**Affiliations:** Instituto Nacional de Cancerología, Mexico City, Distrito Federal, Mexico; INSTITUTO NACIONAL DE CANCEROLOGÍA, MEXICO CITY, Distrito Federal, Mexico; INSTITUTO NACIONAL DE CANCEROLOGÍA, MEXICO CITY, Distrito Federal, Mexico; INSTITUTO NACIONAL DE CANCEROLOGÍA, MEXICO CITY, Distrito Federal, Mexico; Instituto Nacional de Cancerologia, Mexico City, Distrito Federal, Mexico

## Abstract

**Background:**

PLWHIV and cancer have an increased risk of mortality. Previous studies show that a 100 cells/mm^3^ drop in CD4+ T cells following chemotherapy increases the risk of mortality by up to 30%. In Mexico almost 40% of patients have a late diagnosis. The aim of this study is to measure the mortality association between the CD4+ T cell count nadir in PLWHIV and cancer.

**Methods:**

We conducted a retrospective study involving PLWHIV and cancer ≥18 *yo* treated at the cancer and AIDS clinic of the Instituto Nacional de Cancerología in México City from January 01, 2005, to December 31, 2015, that received chemotherapy, immunotherapy, radiotherapy and/or oncologic surgery. The baseline CD4+ T cell count, nadir during treatment, first CD4+ T cell count after treatment, one year and five years after the end of oncologic treatment were evaluated. A logistic regression (LR) model was performed considering CD4+ T cell count at baseline, nadir during oncologic treatment and the association with all-cause mortality. Institutional Review Board (IRB) of INCan approved the study (Ref/INCAN/CI/0049/2022).

**Results:**

A total of 445 patients were included, median of nadir CD4+ T cell count was 122 cells/µL (IQR 48-232), 147 (32.89%) had baseline CD4+ T cell ≤100 cells/µL. Kaposi Sarcoma (46%) and non-Hodgkin lymphoma (27%) were the most frequent diagnosis. One-year mortality occurred in 72 patients (16%), from them, the cause of death was cancer in 54 (75%). Descriptive analysis by mean difference showed significant difference in CD4+ T cell count between patients who died vs. those who survived 117.7 vs 175.4 (p=0.01). In the LR model, a significant association was found between stage IV of cancer and higher mortality at 1 year (OR 2.39; CI 1.27- 4.52) and at 5 years (OR 2.21; CI 1.21-4.02). At five years, mortality was higher in patients with a second AIDS defining event (OR 2.17; CI 1.12-4.17). At one year after completed cancer treatment having a CD4+ T cell nadir >200 cells/ µL was associated with lower mortality (OR 0.29; CI 0.08 – 0.92).

**Conclusion:**

This study found that having other AIDS defining events and advanced neoplastic disease was associated with higher mortality; while having a nadir ≥200 cd4+ cells/ml showed lower mortality after one year of treatment.

**Disclosures:**

**All Authors**: No reported disclosures

